# Changes in Anterior Segment Morphology of Iris Bombe before and after Laser Peripheral Iridotomy in Patients with Uveitic Secondary Glaucoma

**DOI:** 10.1155/2016/8496201

**Published:** 2016-10-31

**Authors:** Wakako Ikegawa, Takashi Suzuki, Koji Namiguchi, Shiro Mizoue, Atsushi Shiraishi, Yuichi Ohashi

**Affiliations:** Department of Ophthalmology, Graduate School of Medicine, Ehime University, Toon, Ehime 791-0295, Japan

## Abstract

*Purpose*. To quantify changes in anterior segment (AS) parameters after laser peripheral iridotomy (LPI) using AS-optical coherence tomography (OCT) of iris bombe.* Method*. AS images of eight eyes were captured before and after iris bombe and more than 2 weeks after LPI (post-LPI) using AS-OCT. We compared the following AS parameters: anterior chamber depth (ACD), anterior chamber volume (ACV), iris curvature (IC), iris thickness at 500 *μ*m from the scleral spur (IT-1) in the middle between the iris root and pupillary margin (IT-2) and 500 *μ*m from the pupillary margin (IT-3) to the anterior chamber angle (ACA) (angle opening distance [AOD750]), and trabecular iris space area.* Results*. Mean IT-1 and IT-3, but not IT-2, were lower after iris bombe (IT-1, *P* = 0.001; IT-2, *P* = 0.081; and IT-3, *P* = 0.001). There were no significant differences between ACD at pre-LPI and before iris bombe (*P* = 0.096). The mean ACV and AOD750 of iris bombe increased at post-LPI (ACV, *P* < 0.01, and AOD750, *P* < 0.05). The mean IT-1, IT-2, and IT-3 increased at post-LPI (all, *P* ≤ 0.01). IC decreased at post-LPI (*P* < 0.001), and ACD at post-LPI did not change.* Conclusions*. The iris extends and becomes thinner during iris bombe. LPI during bombe decreases the IC and increases the ACV and ACA.

## 1. Introduction

Uveitis causes glaucoma, cataract, and retinal damage, resulting in severe visual loss. Uveitic glaucoma includes iris bombe that is an uncommon severe complication of uveitis [1]. Iris bombe is induced by formation of the posterior synechiae and pupillary block. Because intraocular pressure (IOP) elevation during iris bombe is caused by angle closure, iridotomy is a treatment option for the reduction of IOP. However, laser iridotomy has a high failure rate as a result of continuous inflammation [2, 3], and little is known about the appropriate treatment for iris bombe. Therefore, to treat this condition, it is necessary to more comprehensively understand the morphology of the iris and anterior segment (AS), to better facilitate bypass of the aqueous outflow.

AS-optical coherence tomography (AS-OCT) has recently been used to visualize AS structures [4]. During this process, customized software measures AS structures including the angles, iris, anterior chamber, and lens. Several studies have described the morphology of the AS in primary angle closure glaucoma [5–8]. Xu et al. reported that there was a significant association between shallow anterior chamber depth (ACD) and the presence of angle closure glaucoma [8]. Miki et al. reported AS-OCT images in two cases of iris bombe before and after laser iridotomy [9]. However, little is known about the changes that occur in AS-OCT parameters in iris bombe before and after laser iridotomy.

Therefore, the aim of the present study was to analyze AS-OCT parameters during iris bombe to better understand the morphology of the angles, iris, anterior chamber, and lens during this disorder.

## 2. Materials and Methods

### 2.1. Study Participants

Eight eyes with iris bombe from seven patients were enrolled in this study. Patient information is shown in Table 1. The mean age was 59 ± 18.6 years. All seven participants were female and were treated with topical steroids before iris bombe. The diagnosis of iris bombe was made in eyes with angle closure, posterior synechiae, and an IOP elevation ≥ 5 mmHg. Laser peripheral iridotomy (LPI) was performed in the superior region of the iris (from the 10 o'clock to the 2 o'clock position) using topical anesthesia with sequential argon (800–1,000 mW, 50 millis, 50 *μ*m, and 10–50 shots) and a Nd:Yag laser at 1–3 mJ (2–10 pulses). Dexamethasone (0.1%) eye drops were administered 4–6 times a day after LPI. IOP was measured in eyes with iris bombe, before iris bombe, and >2 weeks after LPI. This study was approved by the Ehime University Review Board and adhered to the tenets of the Declaration of Helsinki. Written informed consent was obtained from each study participant.

### 2.2. AS-OCT Imaging

**Figure 1:**
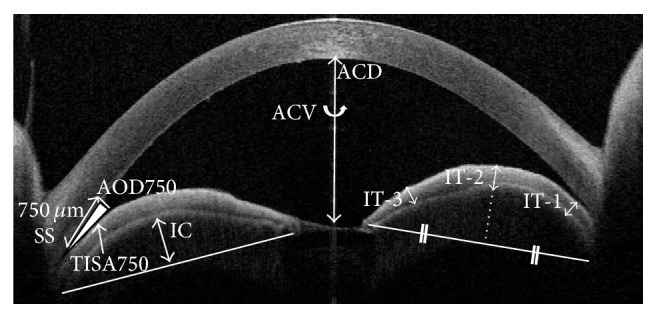
Anterior segment optical coherence tomography (AS-OCT) of iris bombe showing where the study variables were measured. SS: scleral spur.

Each eye was imaged using AS-OCT (swept-source 1000 Casia AS-OCT; Tomey, Nagoya, Japan) by an experienced operator who was masked to the results of the ophthalmic examinations. This AS-OCT system used a 1,310 nm wavelength coupled with high resolution to deliver 30,000 A scans per second with an axial resolution <10 *μ*m that could scan the entire AS in one frame [10]. Imaging by AS-OCT was performed for all of the subjects under room light conditions of 990 lux. Images of the anterior chamber angle (ACA) were obtained using AS-OCT at the temporal and nasal angles of the anterior chamber in the horizontal meridian. All of the images were processed and analyzed by two graders (Wakako Ikegawa and Takashi Suzuki) who were masked to the demographics of the subjects. The two graders agreed on their evaluations of study parameters. The instrument software in the SS-1000 Casia instrument analyzed the following parameters: anterior chamber depth (ACD; mm), anterior chamber volume (ACV; mm^3^), ACA (angle opening distance [AOD750; mm] and trabecular iris space area [TISA750; mm^2^]), iris thickness (IT; mm), iris volume (IV; mm^3^), and iris curvature (IC; mm) [11]. The AOD750 was defined as the distance from the corneal endothelium to the anterior iris surface, perpendicular to a line drawn 750 *μ*m anterior to the scleral spur. The TISA750 was defined as the trapezoidal area with the following boundaries: anteriorly, the AOD750; posteriorly, a line drawn from the scleral spur perpendicular to the plane of the inner scleral wall opposing the iris; superiorly, the inner corneoscleral wall; and inferiorly, the iris surface. The IC was defined as the maximum distance from the posterior boundary of the iris and iris root. The IT was measured at 500 *μ*m from the scleral spur (IT-1), in the middle between the iris root and pupillary margin (IT-2), and 500 *μ*m from the pupillary margin (IT-3; mm) (Figure 1). The ACD, ACV, AOD750, TISA750, and IV were automatically calculated by the internal software of the instrument. The ACD, AOD750, TISA750, IT, and IC were determined using AS-OCT images that were acquired in the horizontal meridian only. The AOD750, TISA750, IT (IT-1, IT-2, and IT-3), and IC were determined as the average of temporal and nasal values. AS-OCT analysis in each eye was performed with angle closure (attack) just after LPI (LPI) and >2 weeks after LPI (post-LPI). Six of seven patients were imaged before iris bombe (preattack).

### 2.3. Statistical Analyses

All of the data are expressed as the mean ± standard deviation (SD). Comparisons of parameters were evaluated by paired *t*-tests. A *P* value < 0.05 was considered statistically significant. Data were analyzed with StatMate IV software (ATMS, Tokyo, Japan).

## 3. Results

**Figure 2:**
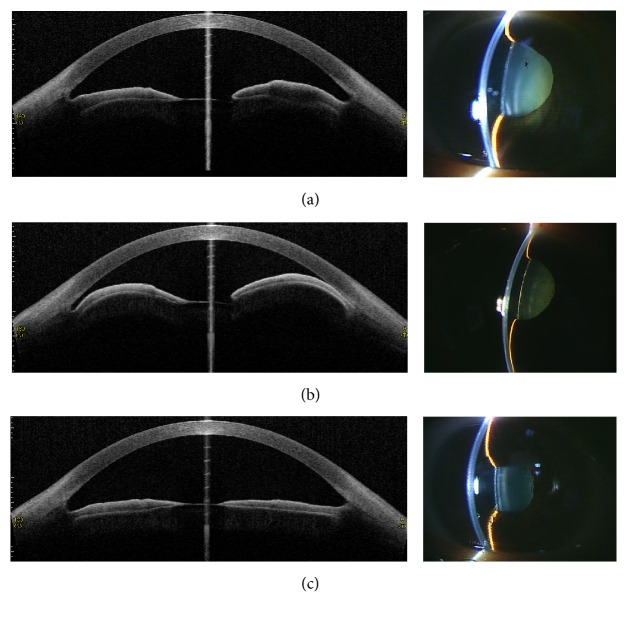
Representative anterior segment optical coherence tomography (AS-OCT) images (left) and slit-lamp images (right) showing (a) preattack, (b) attack, and (c) post-LPI.

All of the subjects had a bypass of aqueous outflow after LPI and resolved angle closure. Although all of the cases did not show a recurrence of iris bombe due to reclosure of the iridotomy window until 2 weeks, two eyes needed cataract surgery due to reclosure of the iridotomy window by fibrin formation after 2 weeks. AS-OCT imaging showed marked iris elevation in the horizontal image and angle closure. Flattening of the iris and opening of the angle were observed immediately after LPI. Representative images are shown in Figure 2. We compared the IOP and AS-OCT parameters at the preattack, attack, immediately after post-LPI, and post-LPI (Table 2). Mean ACV, AOD750, IT-1, and IT-3 significantly decreased when assessed at attack. Moreover, IC significantly increased at attack. However, there was no significant change in ACD, IT-2, or IV. Mean ACV, AOD750, TISA750, IT-1, IT-2, IT-3, and IV significantly increased, and IC significantly decreased immediately after post-LPI. ACV, AOD750, and IT (IT-1, IT-2, and IT-3) significantly increased at post-LPI. IOP significantly increased at attack and decreased at post-LPI.

## 4. Discussion

Iris bombe in uveitic eyes is a unique pathology of acute angle closure. Pupillary block induced by inflammation in the AS is a trigger for this disorder. However, little is known about the morphological changes in the AS during iris bombe. In the present study, AS-OCT showed that AS parameters including ACV, AOD750, IT-1, and IT-3 decreased during iris bombe, compared to preattack, but recovered after LPI. These changes are similar to previous studies of acute angle closure crisis (ACCC) [5, 12]. Lee et al. reported that eyes with ACCC had a shallower ACD and smaller ACA compared to fellow eyes [5]. Moreover, acute attack-affected eyes had a greater lens vault (LV). Acute phacomorphic angle closure is a secondary type of angle closure disorder. Compared to ACCC, Mansouri et al. recently reported that phacomorphic angle closure resulted in a shallower ACD and greater LV, axial length, and ACA [12]. In contrast to ACCC or phacomorphic angle closure, iris bombe does not involve a shallow ACD. Furthermore, the IC during iris bombe was larger compared to previous studies of ACCC [5]. Although ACCC and phacomorphic angle closure could be related to lens parameters, iris bombe is not influenced by the lens. IC is usually used to evaluate relative pupillary block [6, 13]. Thus, iris bombe could be associated with a relative pupillary block without a large anterior movement or swelling of the lens. The iris could be extended by pressure of the posterior chamber, resulting in angle closure. Evaluation of the IC could therefore be used to estimate the relative pupillary block during iris bombe and could also be used as an indicator for LPI.

The root of the iris (IT-1) and the papillary margin (IT-3), but not the middle of the iris (IT-2), were significantly thinner, compared to values before attack, suggesting that the posterior chamber pressure could extend the iris root and papillary margin, compared to the middle iris. Matsuki et al. reported that there was a negative association between the IT and IC during angle closure [6]. Thus, extension of the iris root and iris bulge could easily induce angle closure.

Type I collagen is present in the basement membrane of iris vessels [14]. A thinner iris with low amounts of type I collagen may be more elastic. Uveitis induces inflammation and damage of the iris that may reduce collagen levels and facilitate iris extension.

The present study showed that iris morphology could recover after LPI, with increasing TISA and AOD, decreasing IC, and a reduction of IOP in all of the patients. As previously noted, iris bombe may not be related to any lens parameters. A previous study reported that iris thickness was associated with a greater decrease in IC and an increase in TISA after LPI [15]. Thus, LPI can release pupillary block. However, two eyes needed cataract surgery due to reclosure of the iridotomy window by fibrin formation. To prevent recurrence of iris bombe, anti-inflammatory treatments should therefore be continued after LPI.

Statistical comparisons of small samples using the *t*-test may be associated with type 1 errors. Therefore, studies with larger sample sizes are needed.

## 5. Conclusions

Iris bombe disorder results in angle closure without a shallower ACD, which may be related to a relative pupillary block. The increased IC in iris bombe may be associated with reversibility of AS changes after LPI.

## Figures and Tables

**Table 1:** Summary of data on seven patients with iris bombe.

Case	Gender	Age at onset (years)	Eye(s) with iris bombe	Causative agent of uveitis (systematic disease)	Recurrence of iris bombe (+/−)
1	F	36	OS	Idiopathic uveitis	−
2	F	68	OU	Systemic lupus erythematosus	OD+ OS−
3	F	30	OD	Idiopathic uveitis	−
4	F	66	OD	Idiopathic uveitis	−
5	F	80	OS	Rheumatoid arthritis	+
6	F	70	OD	Exogenous endophthalmitis	−
7	F	63	OD	Rheumatoid arthritis	−

**Table 2:** Comparison of AS parameters among preattack, attack, immed-post-LPI, and post-LPI evaluations.

	Preattack	Attack	Immed-post-LPI	Post-LPI	*P* values			
					Preattack versus attack	Attack versus immed-post-LPI	Attack versus post-LPI	Preattack versus post-LPI
ACD (mm)	2.37 ± 0.22	2.17 ± 0.30	2.28 ± 0.22	2.52 ± 1.07	0.096	0.139	0.112	0.296
ACV (mm^3^)	106.77 ± 27.80	59.66 ± 16.53	110.17 ± 28.47	126.46 ± 28.27	0.01	0.002	0.004	0.141
ACA								
AOD750 (mm)	0.33 ± 0.11	0.10 ± 0.14	0.35 ± 0.19	0.69 ± 0.34	0.02	0.012	0.029	0.107
TISA750 (mm^2^)	0.19 ± 0.06	0.07 ± 0.09	0.17 ± 0.09	0.33 ± 0.19	0.057	0.02	0.053	0.168
IT								
IT-1 (mm)	0.39 ± 0.07	0.25 ± 0.04	0.32 ± 0.04	0.37 ± 0.07	0.001	0.003	0.009	0.854
IT-2 (mm)	0.43 ± 0.15	0.32 ± 0.06	0.38 ± 0.07	0.39 ± 0.05	0.081	0.029	0.006	0.652
IT-3 (mm)	0.35 ± 0.22	0.29 ± 0.04	0.34 ± 0.05	0.33 ± 0.14	0.001	0.012	0.01	0.089
IV (mm^3^)	33.58 ± 22.19	27.34 ± 7.71	42.73 ± 20.27	35.44 ± 20.26	0.793	0.015	0.211	0.329
IC (mm)	0.23 ± 0.17	0.72 ± 0.17	0.22 ± 0.14	0.18 ± 0.09	<0.001	<0.001	<0.001	0.233
IOP (mmHg)	15.33 ± 14.75	26.50 ± 14.76	21.38 ± 11.30	16 ± 9	0.016	0.265	0.035	0.749
